# CTRP1 Knockout Attenuates Tumor Progression in A549 and HCT116 Cancer Cells

**DOI:** 10.3390/cancers14184495

**Published:** 2022-09-16

**Authors:** Rackhyun Park, Yea-In Park, Yeonjeong Park, Siyun Lee, Jaeyeon So, Junsoo Park

**Affiliations:** 1 Division of Biological Science and Technology, Yonsei University, Wonju 29493, Korea; 2 Division of Biological Sciences, Yong-In University, Yongin 17092, Korea

**Keywords:** CTRP1, NF-κB, metastasis, tumor progression

## Abstract

**Simple Summary:**

CTRP1 belongs to the C1q and TNF-related protein family, and we generated CTRP1 knockout cells to examine the role of CTRP1 in tumor progression. CTRP1 knockout attenuates cell growth, invasion and tumor growth in mice, suggesting that CTRP1 expression promotes tumor progression.

**Abstract:**

C1q and TNF-related 1 (C1QTNF1/CTRP1) is an adiponectin-associated protein belonging to the C1q/TNF-related protein family. Recent studies have shown that the C1q and TNF-related protein (CTRP) family is involved in cancer progression; however, the specific role of CTRP1 in tumor progression has not yet been elucidated. To examine the role of CTRP1 in tumor progression, we generated CTRP1 knockout A549 and HCT116 cell lines, which reduced the expression levels of nuclear factor (NF)-κB-dependent and metastasis-promoting transcripts. We demonstrated that CTRP1 knockout inhibited the cell proliferation and invasion and tumor growth. Finally, database analysis showed that CTRP1 expression was upregulated in metastatic cancers and elevated levels of CTRP1 were associated with poor prognosis. These results suggest that CTRP1 expression contributes to NF-κB signaling and promotes tumor progression.

## 1. Introduction

Nuclear factor (NF)-κB is one of the major transcription factors involved in immune and inflammatory mechanisms [1,2,3]. NF-κB is a well-known tumor promoter that regulates all stages of tumor development, including tumor initiation and progression [4,5]. NF-κB is also involved in tumor resistance to chemotherapy and radiotherapy [6,7]. Constitutive activation of NF-κB contributes to tumor cell growth and proliferation [8,9]. Moreover, NF-κB activation promotes while its inhibition suppresses cancer metastasis [8,9,10,11]. For example, the loss of E-cadherin is known to promote cancer metastasis, and the activation of NF-κB reduces E-cadherin expression, which suggests the involvement of NF-κB in cancer metastasis [12,13].

The C1q and TNF-related protein (CTRP) family contains adiponectin and additional 15 CTRP proteins, which are associated with metabolism and immunity [14,15,16]. For example, adiponectin secretion activates the oxidation of fatty acids in the skeletal muscle and reduces the formation of glucose in the liver for homeostasis [17]. CTRP family proteins play important roles in the progression of various cancers, including liver, lung, and colon cancers [18]. As a member of the CTRP family, CTRP1 is mainly expressed in adipose tissue, and a high body mass index correlates with high plasma levels of CTRP1 [19,20]. Recently, we showed that the secreted form of CTRP1 activates cancer cell proliferation via the p53-dependent pathway, and another study identified CTRP1 as a prognostic biomarker in human glioblastoma [21,22]. CTRP1 is also known to activate AMP-activated protein kinase-dependent and extracellular signal-regulated kinase (ERK) signaling pathways [21,23]. However, the role of CTRP1 in tumor progression has not yet been fully elucidated.

In this study, we generated CTRP1 knockout A549 and HCT116 cells and examined the role of CTRP1 in cancer progression. We also evaluated the effect of CTRP1 on tumor progression by examining its expression in metastatic tumors and its association with prognosis.

## 2. Materials and Methods

### 2.1. Cell Culture and Proliferation Assay

A549 human lung cancer cells and HCT116 human colon cancer cells were maintained in Dulbecco’s Modified Eagle’s Medium (Welgene, Seoul, Korea) supplemented with 10% fetal bovine serum (Thermo Fisher Scientific, Waltham, MA, USA) and an antibiotic-antimycotic solution (Welgene). Cell proliferation was measured using the [4,5-dimethylthiazol-2-yl]-2,5-di-phenyltrazolium bromide (MTT) assay. Briefly, cells were seeded in a 24-well plate and incubated overnight. At the indicated time points, MTT solution was added to a final concentration of 1 mg/mL and incubated for 3 h. MTT was purchased from the USB Corporation (Cleveland, OH, USA).

### 2.2. Generation of Knockout Cell Lines

For CTRP1 knockout cell lines, CTRP1 guide RNAs (gRNAs) were cloned into lentiCRISPR V2 vectors (Addgene, Cambridge, MA, USA) with the following primers: CTRP1 knockout #1 sgRNA forward sequence 5′-CACCGAAGATGGGCTCCCGTGGAC-3′ and reverse sequence 5′-AAACGTCCACGGGAGCCCATCTTC-3′; CTRP1 knockout #2 sgRNA forward sequence 5′-CACCGTCGGCATGGTCCGGAGGCGA-3′ and reverse sequence 5′-AAACTCGCCTCCGGACCATGCCGAC-3′. Each forward and reverse oligonucleotide was incubated in the GeneAmp 2720 Thermal Cycler (Thermo Fisher Scientific) for the annealing of double-stranded oligos. Double-stranded oligos cloned into the lentiCRISPR v2 vector were cleaved using Esp3I (BsmBI) (Thermo Fisher Scientific).

### 2.3. Virus Production and Transduction

Lentiviruses were produced via the co-transfection of the lentiviral transfer vector with the psPAX2 envelope and pMD2.G packaging plasmids into HEK293T cells using the calcium phosphate transfection method (2 M CaCl_2_, 2X HEPES buffered saline [pH 7.2]). The medium was changed 12 h after transfection. The virus-containing supernatant media were collected 48 and 72 h after transfection and passed through a 0.45 µm filter. The lentivirus was concentrated using a Lenti-X-concentrator (Clontech, Mountain View, CA, USA) in accordance with the manufacturer’s instructions. Cells in a 6-well tissue culture plate were infected with media containing 8 µg/mL polybrene. At 24 h post-infection, the media was changed, and cells were selected with puromycin (2 µg/mL). Control cells were generated by transduction of an empty vector in similar fashion.

### 2.4. Western Blotting

For protein immunoblotting analysis, polypeptides in whole cell lysates were resolved using sodium dodecyl sulfate-polyacrylamide gel electrophoresis and transferred onto polyvinylidene fluoride membrane filters (Bio-Rad, Hercules, CA, USA). Proteins were detected with 1:1000 or 1:5000 dilution of the primary antibody using an enhanced chemiluminescence system (Dogen, Seoul, Korea). Images were acquired using the LAS4000 system (GE Healthcare, Uppsala, Sweden). The CTRP1 antibody was purchased from Invitrogen (Carlsbad, CA, USA), and p65 antibody was purchased from Cell Signaling Technology (Danvers, MA, USA).

### 2.5. Quantitative Reverse Transcription-Polymerase Chain Reaction (qRT-PCR)

For qRT-PCR, cells were harvested, and RNA was extracted using TRIzol (Invitrogen), according to the manufacturer’s instructions, and subjected to RT-PCR using the StepOnePlus Real-Time PCR System (Applied Biosystems, Foster City, CA, USA). *NF-κB* mRNA was amplified using the forward primer 5′-ACTGTTCCCCCTCATCTTCC-3′ and reverse primer 5′-TGGTCCTGTGTAGCCATTGA-3′. *TNF-α* mRNA was amplified using the forward primer 5′-CTCTTCTGCCTGCTGCACTTTG-3′ and reverse primer 5′-ATGGGCTACAGGCTTGTCACTC-3′. Intercellular adhesion molecule 1 (*ICAM-1*) mRNA was amplified using the forward primer 5′-GGCCGGCCAGCTTATACACA-3′ and reverse primer 5′-TAGACACTTGAGCTCGGGCA-3′. Inducible nitric oxide synthase (*iNOS*) mRNA was amplified using the forward primer 5′-TGGATGCAACCCCATTGTC-3′ and reverse primer 5′-CCCGCTGCCCCAGTTT-3′. Cyclooxygenase 2 (*COX2*) mRNA was amplified using the forward primer 5′-ATCATTCACCAGGCAAATTGC-3′ and reverse primer 5′-GGCTTCAGCATAAAGCGTTTG-3′. The input RNA was normalized via the amplification of ribosomal protein L4 RNA with the forward primer 5′-GCTCTGGCCAGGGTGCTTTTG-3′ and reverse primer 5′-ATGGCGTATCGTTTTTGGGTTGT-3′.

### 2.6. Transwell Invasion Assay

For the Transwell invasion assay, mixture (Matrigel Basement Membrane Matrix (Corning, NY, USA) was added and diluted with serum-Free DMEM media) into the upper compartment of the insert and incubated at 37 °C for 1 h. After incubation, cells with serum-free DMEM media were seeded into the upper compartment of the insert and DMEM supplemented with 10% FBS was added to the well of the plate (lower compartment) as an attractant. The plate was incubated at 37 °C for 48 h and fixed the cells on the lower side of the insert membrane with 4% paraformaldehyde for 10 min and stained with 0.5% crystal violet. Images were captured using a BX53 microscope (Olympus, Tokyo, Japan). The Transwell plate was purchased from Thermo Fisher Scientific (Waltham, MA, USA).

### 2.7. Hematoxylin and Eosin (H&E) Staining

For H&E staining, the tissues were collected, fixed with paraformaldehyde, and embedded in paraffin at room temperature on a microtome in accordance with the manufacturer’s instructions. After paraffinization, the sections were deparaffinized with xylene and rehydrated with ethanol and ddH_2_O. After the rehydration step, the tissues were treated with hematoxylin nuclear stain and excess background stain was removed. After hematoxylin staining, eosin was applied as a counterstain and washed using tap water. Images were captured using a BX53 microscope.

### 2.8. Tumor Xenograft

Six-week-old BALB/c-nu mice were purchased from Raonbio (Seoul, Korea). Mice were injected with 10^7^ cells of control LentiV2 or CTRP1 knockout clones. After injection, the tumor dimensions were measured using a caliper and the volume was calculated using the formula V = (W2 × L)/2.

### 2.9. Statistical Analysis

The results of the western blotting, transmission electron microscopy data, and LC3 puncta analysis were evaluated by a two-tailed *t*-test using Excel software (Microsoft, Seattle, WA, USA). Statistical significance was set at *p* < 0.05.

## 3. Results

### 3.1. CTRP1 Knockout Decreases NF-κB and NF-κB-Dependent Transcript Levels

**Figure 1:**
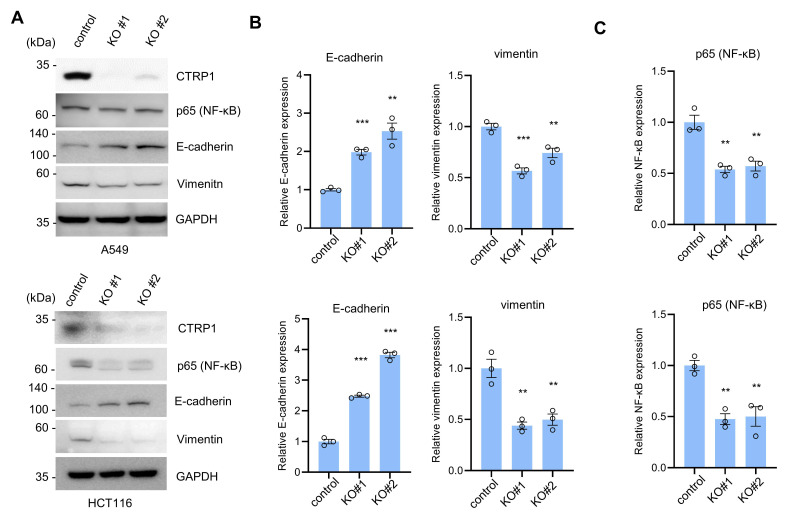
C1q and TNF-related 1 (C1QTNF1/CTRP1) knockout deregulates metastasis marker and nuclear factor (NF)-κB protein. (**A**) CTRP1 knockout cell lines were generated using the clustered regularly interspaced palindromic repeat (CRISPR)/CRISPR-associated protein 9 (Cas9) lentivirus in A549 and HCT116, cells. Equal amounts of cell lysates were probed with the indicated antibody. The uncropped blots are shown in the Supplementary Materials File S1. (**B**) Expression levels of metastatic markers were deregulated in CTRP1 knockout cells. E-cadherin protein levels were increased and vimentin protein levels were decreased in CTRP1 knockout cells. The level of each protein in panel (**A**) was quantified and depicted in the graph. Control vs. knockout, ** *p* < 0.01, *** *p* < 0.005. (**C**) The levels of p65 were decreased in CTRP1 knockout cells. Control vs. knockout, ** *p* < 0.01, *** *p* < 0.005.

We generated CTRP1 knockout cells using A549 and HCT116 cells using the clustered regularly interspaced palindromic repeat (CRISPR)/CRISPR-associated protein 9 (Cas9) system, and the expression of CTRP1 was significantly reduced in CTRP1 knockout cells (Figure 1A). To identify the function of CTRP1 in cancer progression, we examined the expression levels of the metastatic markers E-cadherin and vimentin. The loss of E-cadherin and elevated vimentin levels are associated with increased metastatic potential [13,24]. CTRP1 knockout showed increased E-cadherin expression and decreased vimentin expression (Figure 1A,B). These results suggest that CTRP1 expression may contribute to metastasis.

**Figure 2:**
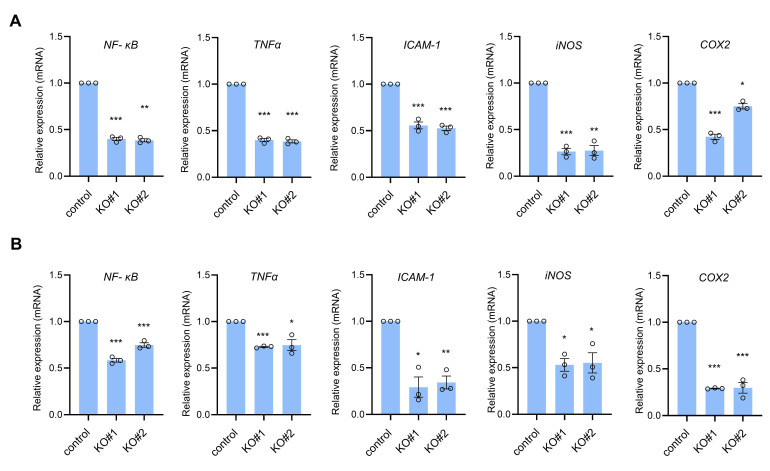
CTRP1 knockout cells show decreased NF-κB expression and NF-κB-dependent transcription. (**A**) mRNA expression levels of NF-κB, TNFα, intercellular adhesion molecule 1 (ICAM-1), inducible nitric oxide synthase (iNOS), and cyclooxygenase 2 (COX2) were measured using quantitative reverse transcription-polymerase chain reaction (qRT-PCR) in A549 cells. (**B**) mRNA expression levels of NF-κB, TNFα, ICAM-1, iNOS, and COX2 were measured using qRT-PCR in HCT116 cells. The means and standard deviations are shown. Control vs. knockout cells: * *p* < 0.05; ** *p* < 0.01; *** *p* < 0.005.

NF-κB signaling is known to promote tumor progression; therefore, we examined the expression of NF-κB signaling in CTRP1 knockout cells. Western blotting data showed that CTRP1 knockout cells had reduced levels of p65, a component of NF-κB, in A549 and HCT116 cells (Figure 1A,C). We also showed that CTRP1 overexpression increased the protein level of p65, and decreased the protein level of E-cadherin in A549 and MCF7 cells (Figure S1). Next, we used quantitative real-time PCR to examine the expression levels of NF-κB- and NF-κB-dependent transcripts induced by CTRP1. We analyzed the mRNA expression levels of NF-κB, TNFα, ICAM-1, iNOS, and COX2 in CTRP1 knockout A549 and HCT116 cells. QRT-PCR showed that CTRP1 knockout resulted in decreased mRNA levels of NF-κB and NF-κB-dependent transcripts (Figure 2A,B). These results suggest that CTRP1 is required for efficient NF-κB-dependent signaling.

### 3.2. CTRP1 Knockout Decreases the Cell Proliferation and Invasion and Cell Cycle Progression

**Figure 3:**
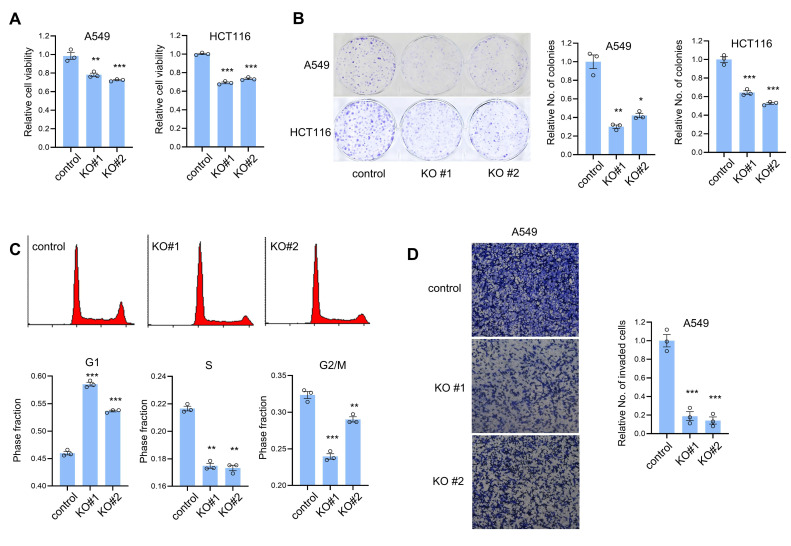
CTRP1 knockout cells show decreased cell proliferation and invasion. (**A**) CTRP1 knockout cells showed decreased cell proliferation. Cell proliferation of control and knockout cells was measured using the 3-(4,5-dimethylthiazol-2-yl)-2,5-diphenyl tetrazolium bromide (MTT) assay. Control vs. knockout cells: ** *p* < 0.01, *** *p* < 0.005. (**B**) CTRP1 knockout decreased colony formation. An equal number of cells (1000 cells) were plated onto the wells of a 6-well plate, and the clonogenic assay was performed for 10 d. The number of colonies was counted and is depicted in the graph (bottom panel). Control vs. knockout cells: * *p* < 0.05, ** *p* < 0.01, *** *p* < 0.005. (**C**) CTRP1 knockout cells showed delayed cell cycle progression. Control and CTRP1 knockout cells were analyzed using flow cytometry and the proportion of G1, S, and G2/M cells were calculated. (**D**) An invasion assay showed that CTRP1 knockout resulted in the decreased invasion ability of A549 cells. Control vs. knockout cells: ** *p* < 0.01, *** *p* < 0.005.

We examined whether CTRP1 affected the cell proliferation using an MTT assay. The cell proliferation assay showed that CTRP1 knockout A549 and HCT116 cells exhibited decreased cell proliferation (Figure 3A). To examine the long-term effect of CTRP1 expression on cell proliferation, we performed a clonogenic assay and found that CTRP1 knockout resulted in fewer countable colonies, indicating that it is required for efficient cell proliferation (Figure 3B). Because CTRP1 knockout decreased cell proliferation, we examined whether CTRP1 expression contributed to cell cycle progression. Flow cytometry data showed that CTRP1 knockout increased G1 phase cells and decreased both S and G2/M phase cells (Figure 3C). Finally, we examined whether CTRP1 contributed to tumor cell invasion using a Transwell invasion assay. CTRP1 knockout cells showed decreased invasion ability in A549 cells (Figure 3D). Collectively, these results indicate that CTRP1 expression contributes to cell proliferation and tumor invasion.

### 3.3. CTRP1 Contributes to Tumor Formation in Mice

**Figure 4:**
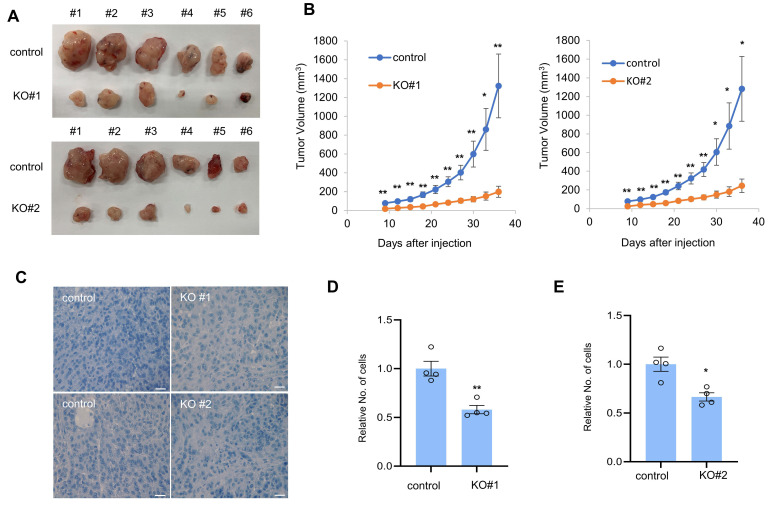
CTRP1 knockout cells show decreased tumor formation in a xenograft mouse model. (**A**) CTRP1 knockout A549 cells did not induce tumors in athymic nude mice. Control and CTRP1 knockout A549 cells were injected in athymic nude mice, *n* = 6. (**B**) CTRP1 knockout A549 cells decreased the tumor size. After injection, tumor volume was measured by a caliper once every three days and shown in the graph. Control vs. knockout cells: * *p* < 0.05, ** *p* < 0.01. (**C**) CTRP1 knockout A549 tumor tissues showed decreased proliferation. Control and knockout tumor tissues were stained with hematoxylin and eosin (H&E), and the number of cells was counted. Control vs. knockout tissues: * *p* < 0.05, ** *p* < 0.01. (**D**) CTRP1 knockout A549 tumor tissues showed decreased NF-κB expression. Each tumor tissue was lysed and subject to western blotting with NF-κB antibody. (**E**) The level of NF-κB protein was quantified and depicted in the graph. Control vs. knockout cells: * *p* < 0.05, ** *p* < 0.01.

Since CTRP1 contributes to cell proliferation and cell cycle progression in vitro, we examined whether CTRP1 contributed to cell proliferation in vivo. To evaluate the role of CTRP1 in vivo, we used a xenograft model of athymic mice and found that CTRP1 knockout A549 cells showed decreased tumor growth in mice (Figure 4A,B). Next, we examined the tumor cell density in tumor tissue. CTRP1 knockout showed decreased cancer cell density in A549 cancer tissues (Figure 4C–E). These results suggest that CTRP1 contributes to tumor growth and formation.

### 3.4. Elevated Expression of CTRP1 Results in Poor Prognosis

**Figure 5:**
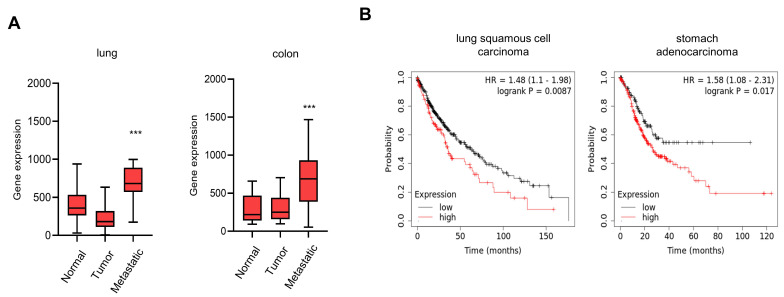
CTRP1 expression is associated with tumor progression. (**A**) mRNA expression of CTRP1 was upregulated in metastatic cancers. The mRNA expression of CTRP1 was analyzed in lung and colon cancers. Nonmetastatic (normal and tumor) vs. metastatic tumor: *** *p* < 0.005. (**B**) Elevated levels of CTRP1 are related with poor prognosis. The high expression of CTRP1 resulted in low survival rates in patients with lung squamous cell carcinoma and stomach adenocarcinoma.

To further identify the association between CTRP1 and tumor progression, we analyzed CTRP1 expression in nonmetastatic (normal and tumor) and metastatic tumors using a TNMplot (https://tnmplot.com/analysis (accessed on 1 May 2022)). While CTRP1 expression was not significantly increased in tumors compared to normal tissues, it was significantly increased in metastatic tumors in colon and lung cancers (Figure 5A and Figure S2). Since metastasis is closely associated with poor prognosis, we analyzed the Kaplan–Meier plots of patients with cancer to determine the relationship between CTRP1 and cancer (https://km.plot/analysis (accessed on 1 May 2022)). Higher CTRP1 expression was associated with poor survival in lung and stomach cancers (Figure 5B). These results suggest that CTRP1 is highly expressed in metastatic cancers and is associated with poor prognosis.

## 4. Discussion

In this study, we demonstrated that CTRP1 contributes to tumor progression. Previously, we showed that treatment of secreted form CTRP1 activates cell proliferation. This study also confirmed that CTRP1 is required for efficient cell proliferation and invasion using CTRP1 knockout cells. Since CTRP1 expression is increased in adipose tissue, studies suggest that obesity is associated with cancer risk [25].

As CTRP1 knockout decreased colony formation and cell proliferation in vitro, we transplanted CTRP1 knockout A549 cells into athymic mice and observed that CTRP1 knockout decreased the tumor formation in vivo. H&E staining data showed that A549 CTRP1 knockout tissues had decreased cell density compared to normal A549 cancer tissues. These results support the hypothesis that CTRP1 is essential for tumor growth in vivo.

We previously showed that the secreted form of CTRP1 activates cell proliferation via the p53-dependent pathway [21]. The secreted form of CTRP1 also contributes to the activation of the ERK signaling pathway. However, we did not find any significant activation of p53 in CTRP1 knockout cells (data not shown). Instead, we found that CTRP1 significantly decreased NF-κB and NF-κB-dependent transcript levels. Although NF-κB signaling contributes to cancer progression, we speculate that multiple signaling pathways are activated by CTRP1. NF-κB is one of the signaling pathways activated by CTRP1, and there may be other signaling pathways contributing to CTRP1-mediated cell growth, invasion, and cancer progression.

Since CTRP1 expression activates cell proliferation and invasion, we speculated that CTRP1 is important for cancer progression. Therefore, we examined the expression levels of CTRP1 in normal, tumor and metastatic tumors by analyzing the gene expression database and found that CTRP1 levels were significantly upregulated in metastatic tumors. Since many patients with cancer die due to metastasis, we examined the relationship between CTRP1 expression and cancer prognosis, and Kaplan–Meier plots showed that elevated levels of CTRP1 in tumors contribute to poor prognosis in lung and stomach cancers. In this study, we used A549 lung cancer and HCT116 colon cancer cells. However, a colon cancer database was not available, so we analyzed the prognosis of lung and stomach cancers.

Since CTRP1 is required for efficient tumor formation, its expression is potentially useful for predicting cancer progression. Elevated levels of CTRP1 in the serum or tumor cells can be used as a marker to examine tumor characteristics. In addition, CTRP1 levels are elevated in metastatic cancers and can be used as a cancer prognostic marker. However, further clinical studies are required to apply CTRP1 as a tumor marker in clinical settings.

## 5. Conclusions

Here, we demonstrate that CTRP1 contributes to tumor progression. We generated CTRP1 knockout cells and CTRP1 knockout attenuates cell proliferation, invasion and tumor growth in mice. CTRP1 knockout also downregulates NF-κB dependent signaling, a major tumor promoting signaling. A database analysis showed that CTRP1 expression is significantly upregulated in metastatic tumors, and the higher expression of CTRP1 is associated with poor prognosis. These results collectively indicate that CTRP1 expression contributes to tumor progression.

## Data Availability

Publicly available datasets were analyzed in this study. This data can be found here: https://tnmplot.com/analysis, https://km.plot/analysis, accessed on 30 July 2022.

## Supplementary Materials

The following supporting information can be downloaded at: https://www.mdpi.com/article/10.3390/cancers14184495/s1, Figure S1. CTRP1 overexpression (OE) increases p65 (NF-κB) protein level and decreases E-cadherin level; Figure S2. mRNA expression of CTRP1 was upregulated in metastatic cancers; File S1. The uncropped blots.

## References

[1] Liu T., Zhang L., Joo D., Sun S.C. (2017). NF-κB signaling in inflammation. Signal Transduct. Target. Ther..

[2] Karin M. (2006). Nuclear factor-kappaB in cancer development and progression. Nature.

[3] Hayden M.S., Ghosh S. (2008). Shared principles in NF-kappaB signaling. Cell.

[4] Cai Z., Tchou-Wong K.M., Rom W.N. (2011). NF-kappaB in lung tumorigenesis. Cancers.

[5] Xia Y., Shen S., Verma I.M. (2014). NF-κB, an active player in human cancers. Cancer Immunol. Res..

[6] Chen W., Li Z., Bai L., Lin Y. (2011). NF-kappaB in lung cancer, a carcinogenesis mediator and a prevention and therapy target. Front. Biosci. (Landmark Ed.).

[7] Solomon L.A., Ali S., Banerjee S., Munkarah A.R., Morris R.T., Sarkar F.H. (2008). Sensitization of ovarian cancer cells to cisplatin by genistein: The role of NF-kappaB. J. Ovarian Res..

[8] Nakshatri H., Bhat-Nakshatri P., Martin D.A., Goulet R.J., Sledge G.W. (1997). Constitutive activation of NF-kappaB during progression of breast cancer to hormone-independent growth. Mol. Cell Biol..

[9] Bargou R.C., Emmerich F., Krappmann D., Bommert K., Mapara M.Y., Arnold W., Royer H.D., Grinstein E., Greiner A., Scheidereit C. (1997). Constitutive nuclear factor-kappaB-RelA activation is required for proliferation and survival of Hodgkin’s disease tumor cells. J. Clin. Investig..

[10] Kawamura I., Morishita R., Tsujimoto S., Manda T., Tomoi M., Tomita N., Goto T., Ogihara T., Kaneda Y. (2001). Intravenous injection of oligodeoxynucleotides to the NF-kappaB binding site inhibits hepatic metastasis of M5076 reticulosarcoma in mice. Gene Ther..

[11] Pires B.R., Mencalha A.L., Ferreira G.M., de Souza W.F., Morgado-Díaz J.A., Maia A.M., Corrêa S., Abdelhay E.S. (2017). NF-kappaB is involved in the regulation of EMT genes in breast cancer cells. PLoS ONE.

[12] Zhang K., Zhaos J., Liu X., Yan B., Chen D., Gao Y., Hu X., Liu S., Zhang D., Zhou C. (2011). Activation of NF-B upregulates Snail and consequent repression of E-cadherin in cholangiocarcinoma cell invasion. Hepato-Gastroenterol..

[13] Onder T.T., Gupta P.B., Mani S.A., Yang J., Lander E.S., Weinberg R.A. (2008). Loss of E-cadherin promotes metastasis via multiple downstream transcriptional pathways. Cancer Res..

[14] Schäffler A., Buechler C. (2012). CTRP family: Linking immunity to metabolism. Trends Endocrinol. Metab..

[15] Seldin M.M., Tan S.Y., Wong G.W. (2014). Metabolic function of the CTRP family of hormones. Rev. Endocr. Metab Disord..

[16] Wong G.W., Krawczyk S.A., Kitidis-Mitrokostas C., Revett T., Gimeno R., Lodish H.F. (2008). Molecular, biochemical and functional characterizations of C1q/TNF family members: Adipose-tissue-selective expression patterns, regulation by PPAR-γ agonist, cysteine-mediated oligomerizations, combinatorial associations and metabolic functions. Biochem. J..

[17] Fang H., Judd R.L. (2018). Adiponectin regulation and function. Compr. Physiol..

[18] Kong M., Gao Y., Guo X., Xie Y., Yu Y. (2021). Role of the CTRP family in tumor development and progression [Review]. Oncol. Lett..

[19] Kim K.Y., Kim H.Y., Kim J.H., Lee C.H., Kim D.H., Lee Y.H., Han S.H., Lim J.S., Cho D.H., Lee M.S. (2006). Tumor necrosis factor-alpha and interleukin-1beta increases CTRP1 expression in adipose tissue. FEBS Lett..

[20] Xin Y., Lyu X., Wang C., Fu Y., Zhang S., Tian C., Li Q., Zhang D. (2014). Elevated circulating levels of CTRP1, a novel adipokine, in diabetic patients. Endocr. J..

[21] Park R., Jang M., Park Y.I., Park Y., Namkoong S., Lee J.I., Park J. (2021). Elevated levels of CTRP1 in obesity contribute to tumor progression in a p53-dependent manner. Cancers.

[22] Chen L., Su G. (2019). Identification of CTRP1 as a prognostic biomarker and oncogene in human glioblastoma. BioMed. Res. Int..

[23] Peterson J.M., Aja S., Wei Z., Wong G.W. (2012). CTRP1 protein enhances fatty acid oxidation via AMP-activated protein kinase (AMPK) activation and acetyl-CoA carboxylase (ACC) inhibition. J. Biol. Chem..

[24] Hu L., Lau S.H., Tzang C.H., Wen J.M., Wang W., Xie D., Huang M., Wang Y., Wu M.C., Huang J.F. (2004). Association of Vimentin Overexpression and hepatocellular carcinoma metastasis. Oncogene.

[25] Roberts D.L., Dive C., Renehan A.G. (2010). Biological mechanisms linking obesity and cancer risk: New perspectives. Annu. Rev. Med..

