# Supplementary material for: CTRP1 Knockout Attenuates Tumor Progression in A549 and HCT116 Cancer Cells
Source: Cancers (Basel). 2022 Sep 16;14(18):4495. doi: 10.3390/cancers14184495 (PMC9496675; doi:10.3390/cancers14184495)
Supplement: Supplementary file 1 [file cancers-14-04495-s001.zip › cancers-1890451-supplementary-figures.pdf]

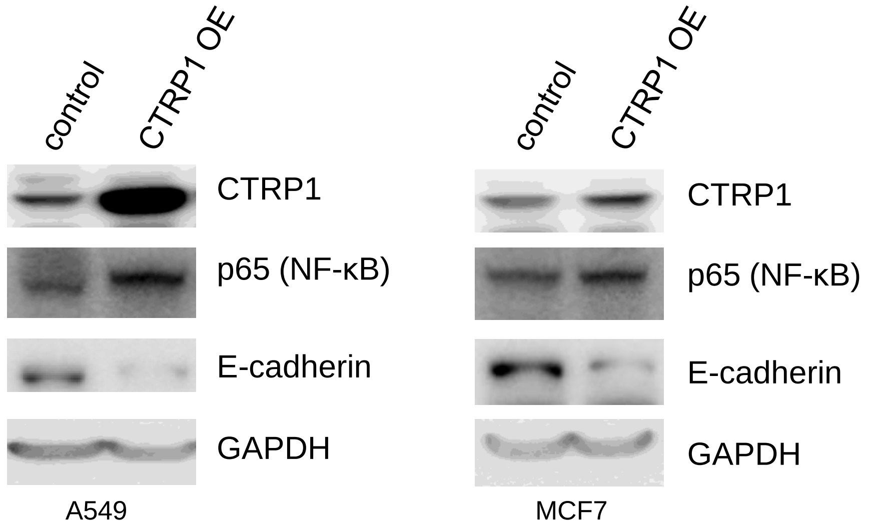

Figure S1. CTRP1 overexpression (OE) increases p65 (NF-κB) protein level and decreases E-cadherin level. A549 Cells and MCF7 cells were infected with either control lentivirus (control) or lentivirus encoding CTRP1 (CTRP1 OE). At 72 h after the infection, cells were selected by puromycin, harvested, and equal amounts of proteins from the cell lysates were probed with the indicated antibodies.

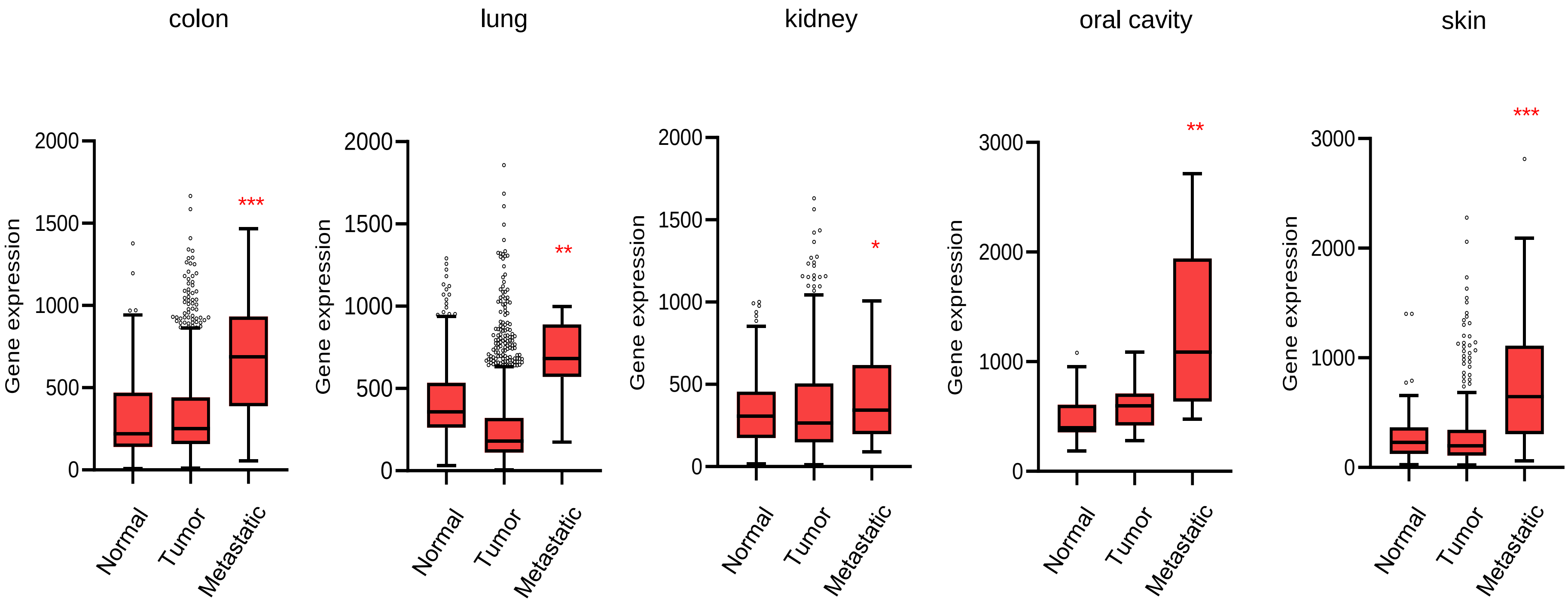

Figure S2. mRNA expression of CTRP1 was upregulated in metastatic cancers. The mRNA expression of CTRP1 was analyzed in colon, lung, kidney, oral cavity and skin cancers. Nonmetastatic (normal and tumor) vs. metastatic tumor: *p < 0.05 , **p < 0.01, ***p < 0.005.
