# Supplementary figures and images for: CTRP1 Knockout Attenuates Tumor Progression in A549 and HCT116 Cancer Cells
Source: Cancers (Basel). 2022 Sep 16;14(18):4495. doi: 10.3390/cancers14184495 (PMC9496675; doi:10.3390/cancers14184495)

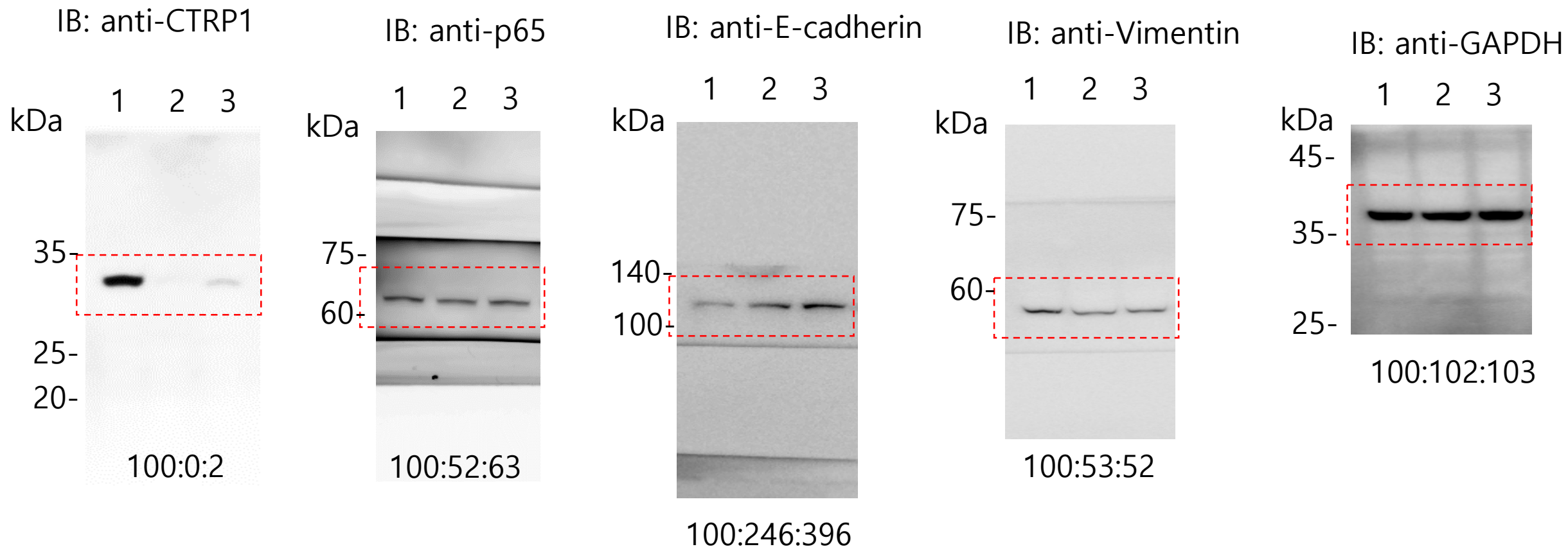

Figure 1A

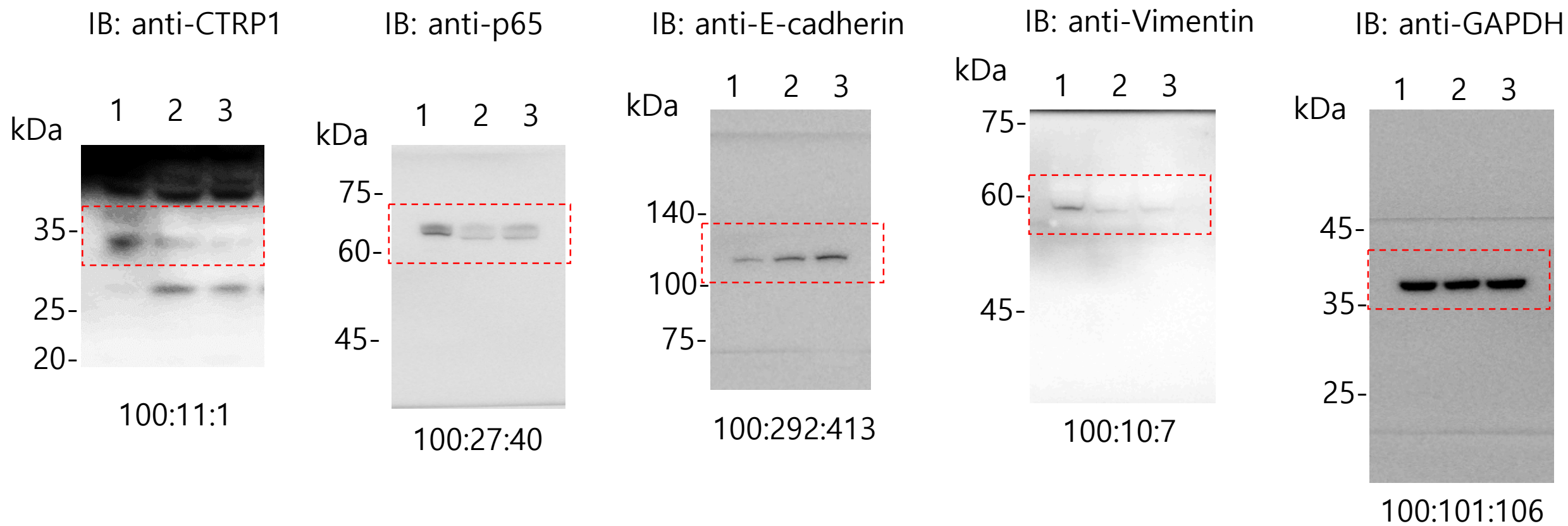

Figure 1A

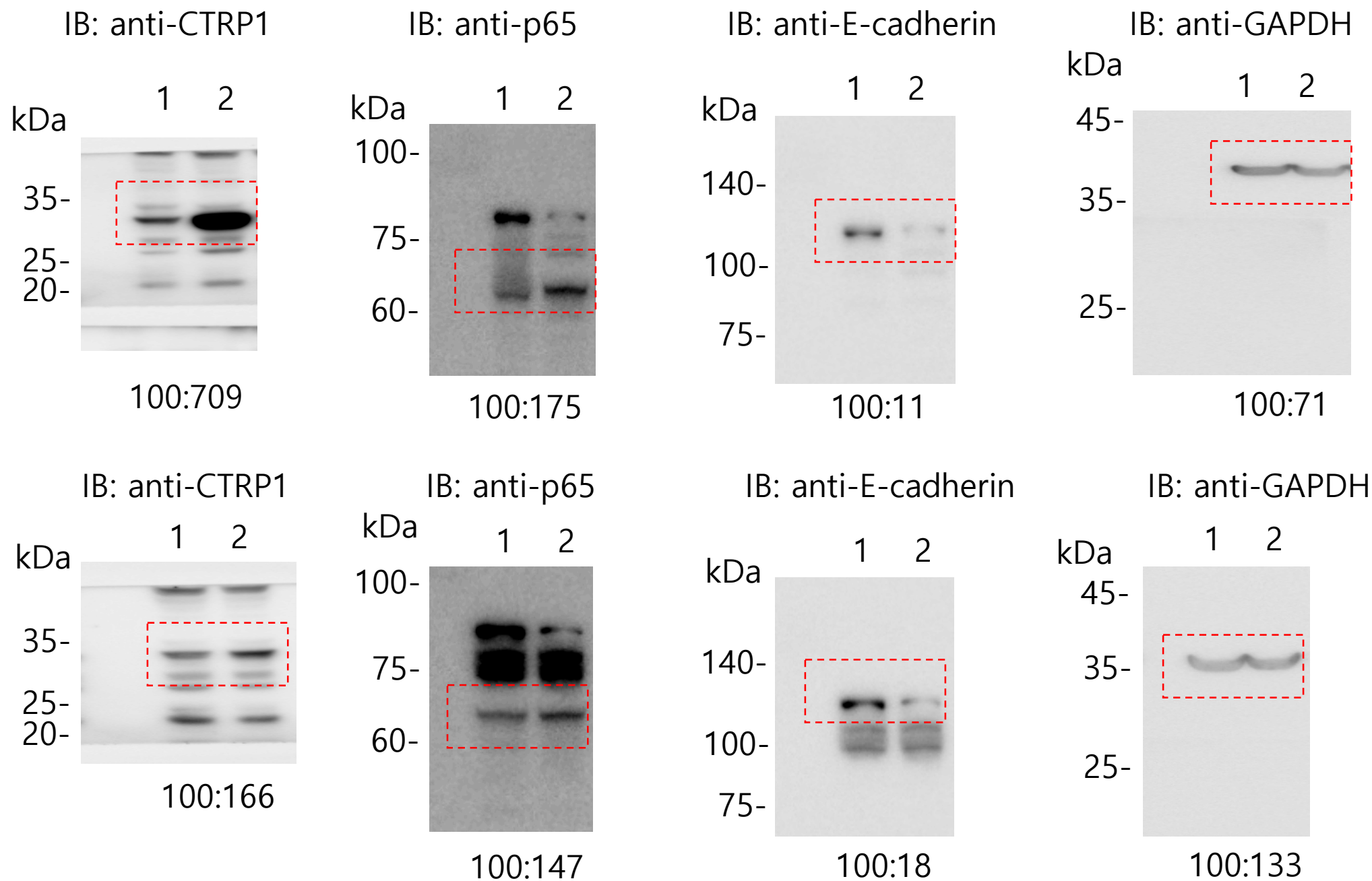

Figure S1

Supplement: Supplementary file 1 [file cancers-14-04495-s001.zip › cancers-1890451-supplementary File S1.pdf]
